# Current Concepts of Ablative Surgery in Oral Cavity Cancer

**DOI:** 10.1007/s12663-024-02188-3

**Published:** 2024-05-11

**Authors:** Yao-Te Tsai, Ku-Hao Fang, Kudav Adarsh

**Affiliations:** 1 Department of Otorhinolaryngology-Head and Neck Surgery, Chang Gung Memorial Hospital, Chiayi, Taiwan; 2 Department of Oral and Maxillofacial Surgery, Manipal College of Dental Sciences, Manipal, Manipal Academy of Higher Education, Manipal, 576104, India

**Keywords:** Oral cancer ablation, Compartment resection, Tumor margins, Resection

## Abstract

**Introduction:**

Ablative surgery has evolved over the years with the attempt to extirpate the tumor in its entirety with the understanding of the molecular tumor biology, pattern of tumor invasion of the tumors, as well as availability of better instrumentations.

**Materials and Methods:**

Subset-based evaluation and management of oral cancer.

**conclusion:**

For oral cancer, surgery is still the primary therapeutic option. To establish surgical adequacy, a wide excision with sufficient margins in all three dimensions must be carried out.

## Introduction

In terms of risk factors, geographic predilections, responsiveness to treatment, and prognosis, oral cavity squamous cell carcinoma (OSCC) is a heterogeneous illness. The first line of treatment for oral malignancies is surgery, which is followed by adjuvant therapy such as radiation or chemoradiation [1].

Ablative surgery has evolved over the years with the attempt to extirpate the tumor in its entirety with the understanding of the molecular tumor biology, pattern of tumor invasion of the tumors, as well as availability of better instrumentations [1].

Surgery is the primary treatment for tumors of the oral cavity in a curative intent setting, although a randomized experiment by Licitra et al. showed that NACT in oral cancers increased the chance of resecting a smaller portion of the mandible [2].

### Preoperative Evaluation

Surgical planning and safe margin acquisition require a thorough understanding of the anatomy and patterns of tumor progression in the oral cavity. Imaging is essential in determining the extent of the disease and the purpose and mode of treatment.

The preferred imaging technique for accessing oral malignancy is contrast-enhanced computed tomography (CECT). Due to its superior soft tissue delineation, magnetic resonance imaging (MRI) is typically advised as an adjuvant. Moreover, it is employed to evaluate perineural invasion, medullary bone involvement, and dural invasion (linear or nodular).

Qiao et al. in their systematic review and metanalysis concluded that CBCT was the top priority choice of imaging methods to diagnose mandibular invasion caused by head and neck cancer. SPECT was recommended as the first option to exclude patients without mandibular invasion, and CT and MRI were suitable for diagnosis conformation [3].

Determining operability requires an accurate assessment of the ITF. For this reason, fat-suppressed T1-weighted post gadolinium MRI images are reported to have greater accuracy for perineural spread and involvement of the skull base foramina, albeit both CECT and MRI are comparable [4].

Lee et all concluded that rDOI measured by US, CT, and MRI showed excellent correlation coefficients with pDOI. For OSCC with advanced T stages, MRI-based DOI measurement would provide more consistent Rdoi [5].

When it comes to negative predictive values, PET-CT is thought to have the greatest, almost reaching 100%. PET-CT is typically reserved for patients with recurrent or second primary disease in a resource-constrained setting, even though according to NCCN recommendations, it has to be indicated for all stage III and IV disease [6].

### Preoperative Evaluation

**Fig. 1:**
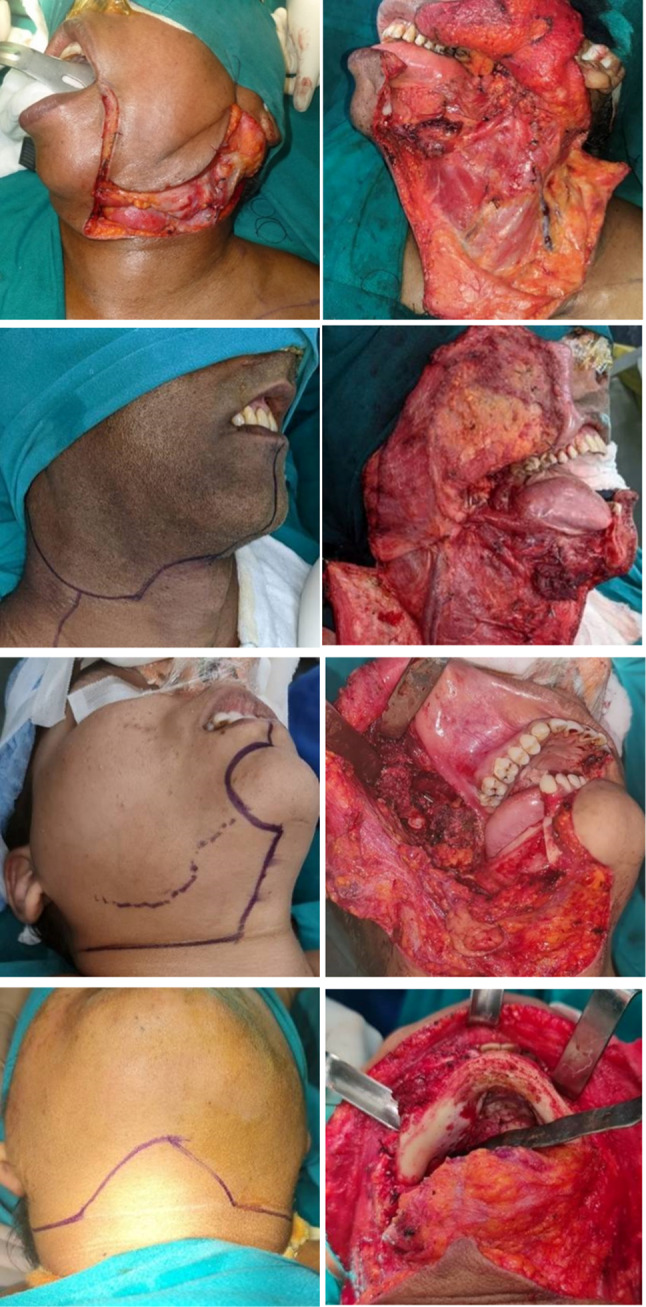
Approaches to mandible. **A** Robson corner split incision, **B** Roux-Trotter incision, **C** McGregor incision, and **D** visor approach

It is critical to recognize and take into account a number of conditions before surgery, such as the following:

1. Incision design for approaching the tumor Fig. 1A–D
2. Whether the mouth opening is adequate
3. Management of the mandible and maxilla
4. Deep soft tissue infiltration
5. Impact of previous resection and reconstruction
6. Possibility of skin sacrifice and related skin wound design
7. Reconstructive option
8. Dental Rehabilitation

Irresectable cancers are tumor invasion into the infratemporal fossa with supra notch involvement, significant soft tissue involvement with induration reaching up to the zygoma, perineural invasion to the foramen ovale or to the trigeminal ganglion, significant tumor extension with great vessel encasement, unresectable nodal disease due to vital structure involvement.

### Principles of Ablative Surgery

1. Adequate access to the tumor.
2. To achieve negative surgical margins.
3. Utilization of intraoperative frozen section for margin assessment.
4. Wide excision versus compartment resection.

### Critical issues Relevant to Each subsite

#### Tongue and Floor of Mouth

**Fig. 2:**
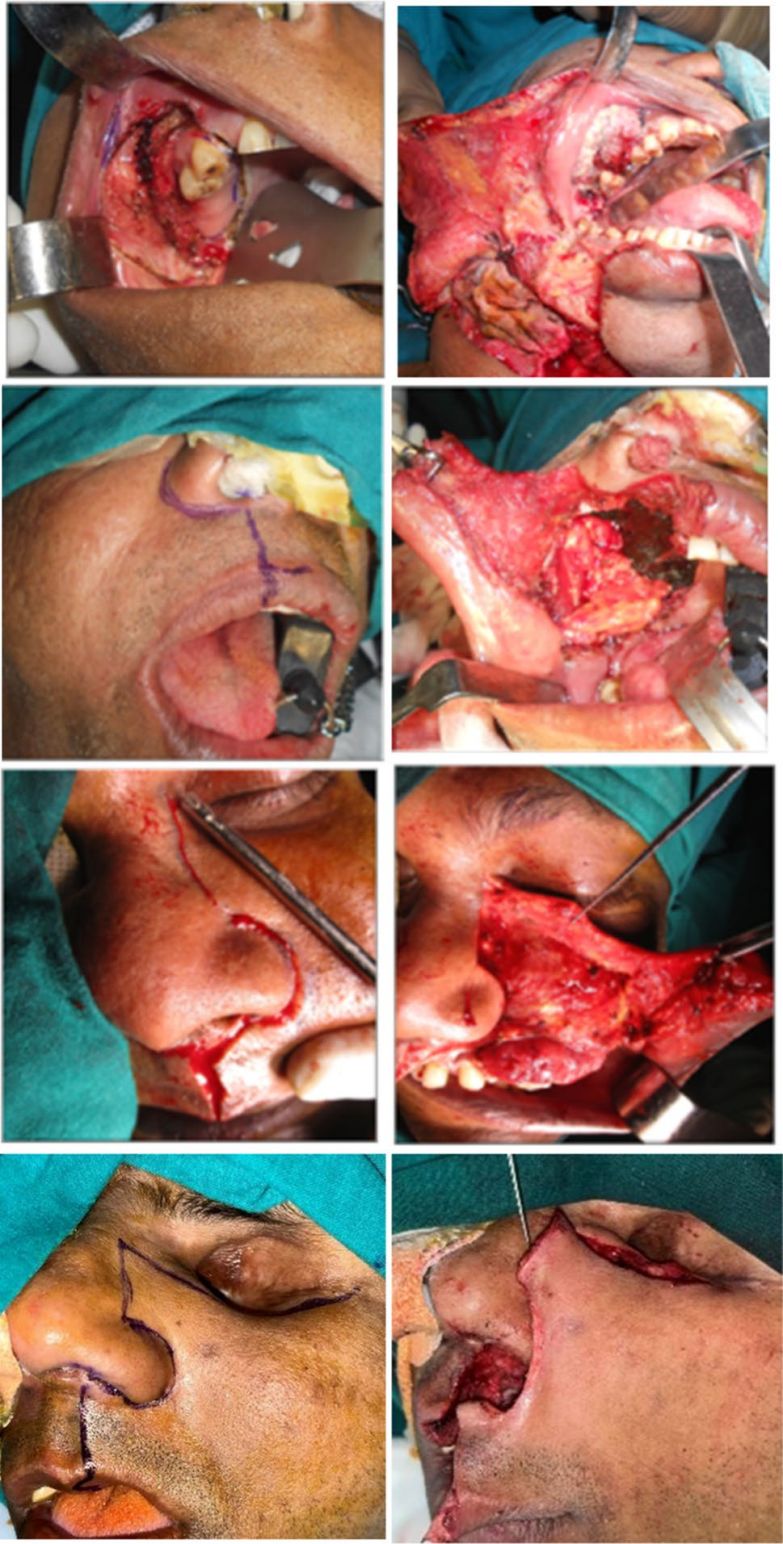
Approaches to maxilla. 1a Per oral approach, 1b-lower cheek flap approach, 2 alotomy, 3 Weber–Fergusons, 4 Dieffenbach’s modification of Weber–Fergusons approach

Perimuscular invasion is the most frequent route via which tongue cancer spreads. The tongue's intricate innervation makes the perineural route one more conceivable method via which oral tongue tumors can spread (Fig. 2).

Wide local excision (WLE)/adequate glossectomy procedure with adequate surgical margins has been the procedure of choice for tongue cancers. Narrow band imaging and intraoperative ultrasonography, vital staining, fluorescent, and chemilumucent technique are adjuncts used during resection.

**Fig. 3:**
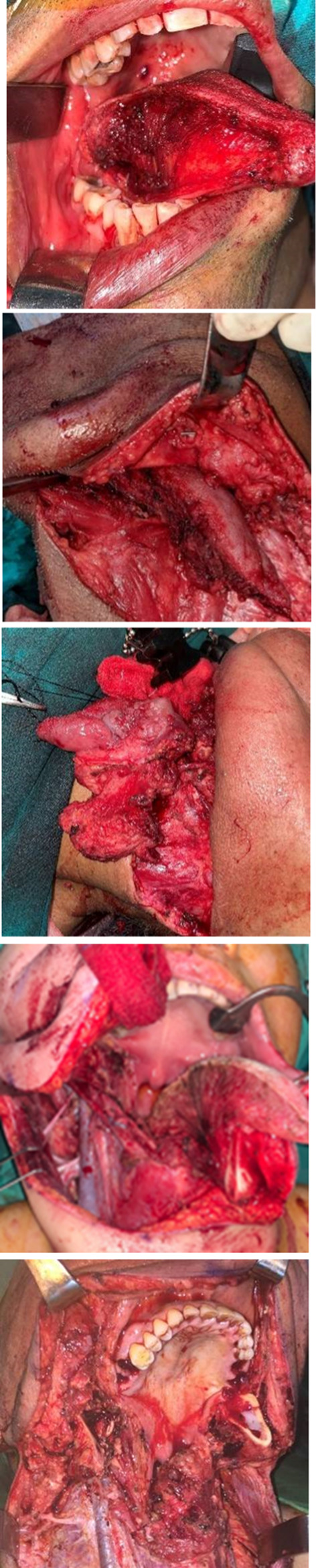
Approaches to tongue. 1 Peroral, 2 lingual release, 3 compartment resection, 4 mandibulotomy approach, and 5 total glossectomy

The traditional approaches described for tongue tumors include intraoral resection, pull-through, mandibular release, mandibulotomy, or a combination of these (Fig. 3a–d).

Ansarin M et al. proposed classification of glossectomy based on surgical anatomy of the tongue which comprises also the routes of spread of the tongue cancer [7].

It is a useful practice to ligate the lingual artery in the neck before performing WLE for adequate bleeding control and clean surgical field (figure).

Choi et al. described Transoral bisected resection for T1-2 oral tongue squamous cell carcinoma to secure adequate deep margin [8].

Rao et al. reported lingual sulcus release is a simple reproducible surgical technique that helps in better visualization of the lateral margin for tongue tumors with trismus [9].

The mandibulotomy is adopted to gain greater exposure in the resection of tumors in the oral cavity. In the original 1836 Roux design, the osteotomy was made between the ‘midline’ central incisors. Later in 1991, Dubner and Spiro proposed the ‘paramedian’ approach in between the lateral incisor and the canine (figure).

A paramedian mandibulotomy preserves the anterior belly of the digastric muscle, the genioglossus, and geniohyoid muscles, minimizing muscle detachment and reducing submental dead spaces. This supposedly results in better tongue movements and a quicker return of swallowing functions [10]. Radiological evidence of the wider dental spaces and root divergence between the lateral incisor and the canine means that the requirement for tooth extraction upon creation of the osteotomy can be avoided, thereby reducing subsequent osteoradionecrosis (ORN). Midline osteotomies also induce the least disruption to the mandibular blood supply, unlike paramedian osteotomies, which rely heavily on the terminal branches of the contralateral inferior alveolar arteries. Theoretically, a paramedian osteotomy has a higher ORN risk for being at the margin of the irradiation field [11].

Chiu et al. concluded that paramedian sites increased the rate of osteoradionecrosis, and correlation with the osteotomy type resulted in more osteoradionecrosis in notched types and more complications in straight paramedian mandibulotomies [12].

Trasmandibular access provided superior local control and DFS compared to TOR in pT2 tongue cancers [13].

Compartmental tongue surgery (CTS) is a surgical technique that removes the compartments containing the primary tumor, eliminating the disease and potential muscular, vascular, glandular, and lymphatic pathways of spread and recurrence. Compartment boundaries are defined as each hemi-tongue bounded by the lingual septum, the stylohyoid ligament and muscle, and the mylohyoid muscle [14].

The markedly improved outcomes in CTS patients, compared to those treated by standard surgery, suggest CTS as an important new approach in the surgical management of tongue cancer [14].

Anatomical unit resection surgery was found to provide a precise surgical treatment to address tongue cancer adjacent to or crossing the midline and maximally maintain tongue tissue and function [15].

The genioglossus is a fan-shaped extrinsic tongue muscle that is derived from the genial tubercles and can be inserted into the hyoid bone and the bottom of the tongue. The genioglossus combined with musculus verticalis linguae forms the majority of the half-tongue body.

Based on the anatomical characteristics of the tongue and AURS, the novel concept of the musculus verticalis linguae–genioglossus complex (MGC) has been developed [15].

The mesial space of the MGC is the lingual septum space, and the outer space of the MGC contains the lingual artery and lingual veins, which highlights the MGC as an anatomical marker for the resection of tongue cancer [15].

AURS provided more precise resection of cancer adjacent to the midline and cancer invading but not breaching the contralateral MGC to maximally preserve tongue function. The ipsilateral MGC served as an anatomical marker for determining the resection of tongue cancer [15].

Locally advanced (T4a) cancers (tumor depth > 20 mm, restricted mobility and hypoglossal palsy) of tongue warrant total glossectomy or near-total glossectomy. Standard total glossectomy procedure involves complete removal of anatomical tongue from mandible to hyoid and from the tip of the tongue up to the vallecula.

### Buccal Mucosa

**Fig. 4:**
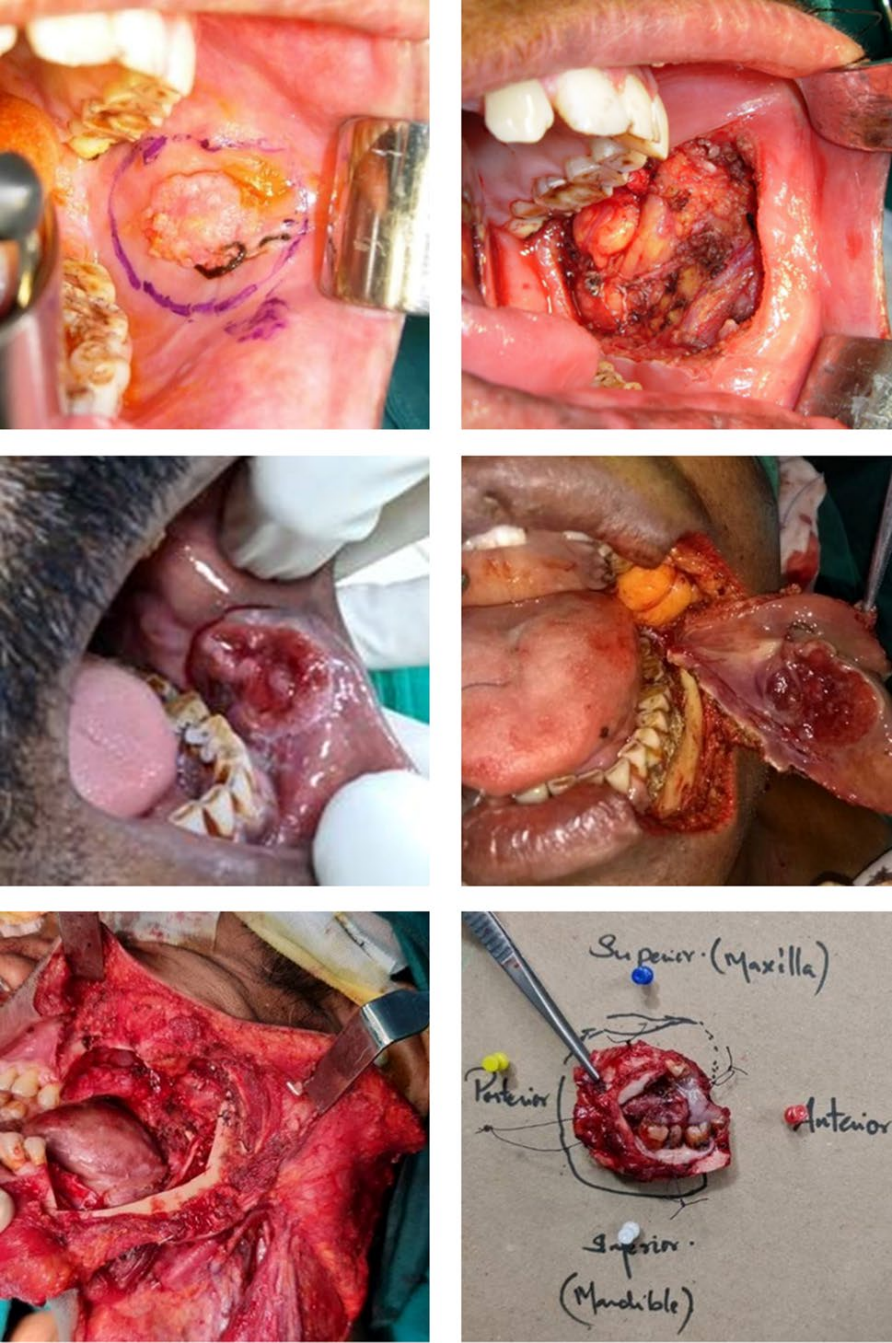
Buccal mucosa approaches. X-Peroral approach, Y-full thickness with skin resection, Z-resection with marginal bone resection

Peroral resection with the buccinator muscle as the deep margin is typically performed for T1 and T2 tumors. Stump repositioning is required when Stensen’s duct is part of the resected specimen. (Fig. 4x–z).

Preoperative clinical examination revealing a subtle skin puckering and imaging studies shows stranding of subcutaneous fat that is the early sign of skin involvement. If buccal space involvement is suspected, then buccal fat pad should be included in the specimen.

These advanced lesions require full-thickness cheek resection. The planning of incision may be a midline lip split or angle split, both of which will help in raising a lower cheek flap or when overlying skin is involved, an incision around the skin involved in continuity with the neck dissection incision. The muscle of mastication involvement warrants infratemporal fossa clearance.

Ren et al. Based on the anatomic characteristics and infiltration of the primary tumor described new surgical approach—unit resection buccal surgery (URBS). Cheek into four parts using the anterior border of masseter and the line of occlusion as the boundary [16].

The concept of compartmental resection was proposed by Trivedi et al. for tumors involving the masticator space. This involved resection of the entire masseter muscle, the medial and lateral pterygoid muscles, the pterygoid plates, part of the temporalis muscle, the mandible, and the soft tissue up to the base of the skull [17].

### Gingivobuccal Sulcus and Mandible

**Fig. 5:**
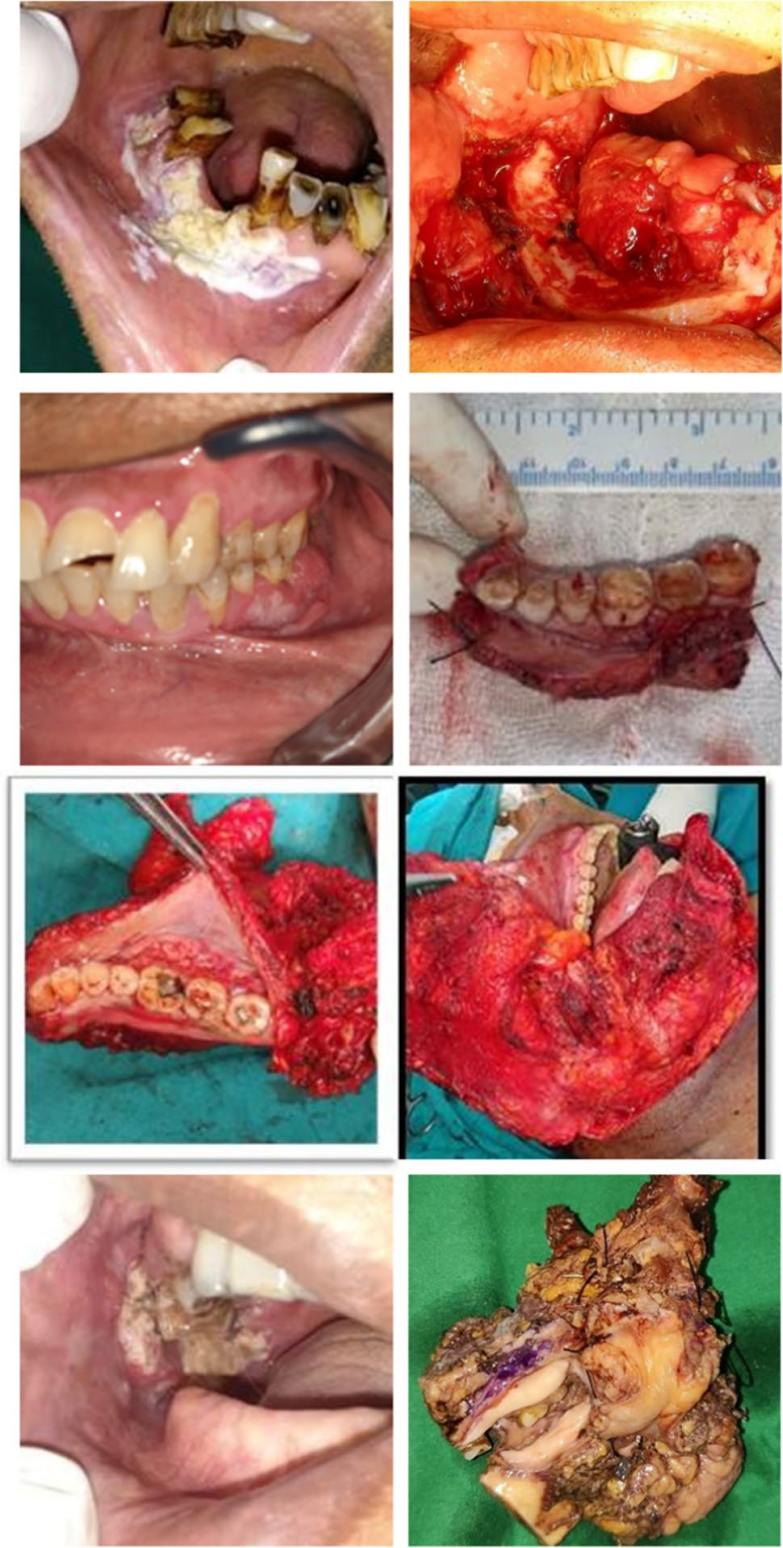
Approaches to mandibular alveolus. **a** Marginal mandibulectomy, **b** segmental mandibulectomy, **c** hemimandibulectomy, and **d** composite resection with infratemporal fossa clearance

Gingivobuccal sulcus (GBS) tumors are tumors occurring in the upper or lower GBS, usually seen to abut the adjacent bone. These occur almost exclusively in Southeast Asia due to high incidence of chewing tobacco use. Due to the high propensity for local invasion and close proximity of bone, skin, and masticator space, presentation is often advanced, and outcomes are poor. If there is superficial erosion of bone or if the lesion is abutting the mandible, then the resection should include marginal mandibulectomy (Fig. 5a–d).

GBS tumors usually present at an advanced stage with gross mandibular erosion, paramandibular spread, or overlying skin involvement. These findings preclude the use of marginal mandibulectomy, and hence patients often require segmental mandibulectomy and bony reconstruction.

Gingivobuccal complex cancers are a homogenous group of cancers with respect to mandibular invasion with a preferential route of tumor entry through the occlusal surface. Large tumors with paramandibular disease are likely to have multiple routes of tumor entry, contraindicating mandibular conservation. Oncological safety can be achieved by positioning the bone cut margin corresponding to the adjacent soft tissue cut margins in segmental mandibulectomy [18].

Segmental mandibular resection is the most important decision to be made in the management of oral cancer. Various classifications have been described, brown et all classification offers an enhanced staging system for this common and challenging surgical problem [19].

From 2010 to 2022, the National Comprehensive Cancer Network (NCCN) treatment guidelines maintain that patients with T4b OCSCC should be treated with either non-surgical modalities or in the context of clinical trials [6].

The AJCC 2017 staging manual, eight edition consider T4b tumors as an expression of very advanced local disease [20].

Evidence has shown selected advanced lesion involving masticatory space which require formal infratemporal fossa clearance have favorable outcome (figure). The tumor excision in T4b tumors should target infra-notch T4b OSCC. For those with infra-notch T4b OSCC, patients with pN0–1, and without nerve invasion may have a favorable outcome than those with either pN2 or pN0–1 with nerve invasion [21].

### Hard palate and Maxillary Gingiva

Approximately 3% and 6% of oral cancers are hard palate and maxillary gingival cancers, respectively.^15^ Although transoral wide excision and resection of the mucoperiosteum that is involved and of underlying bone may be adequate for small maxillary gingival cancer (Fig. 21–4).

Various classifications have been described for maxillectomy defects and its reconstruction, notably browns [22] and Cordeiro [23] are frequently followed.

Lesions of the posterior alveolus and hard palate have a higher tendency to locally invade the orbital floor and skull base or through various neurovascular bundles (greater palatine foramen, sphenopalatine foramen, palatovaginal canal).

Advanced lesions requiring subtotal or total maxillectomy are approached via facial degloving standard or weber Ferguson and its modifications (figure). In cases of posterior extension to the pterygoid region, formal infratemporal clearance is advised. Lower cheek flap approach [24] and mandibulotomy for maxillectomy approaches have been described [25].

However, when the tumor extends to the medial orbital wall, ethmoids or when there is intracranial extension, Weber–Fergusson and bicoronal incisions may be incorporated with the mandibulotomy approach [25].

### Neoadjuvant Chemotherapy

Although mandibular involvement precludes organ preservation strategies, there is a significant subset of patients with tumors in proximity to the mandible, necessitating resection primarily to achieve adequate soft tissue clearance of disease. Randomized controlled trial by Licitra et al. (2011) showed that NACT in oral cancers increased the chance of resecting a smaller portion of the mandible [2].

The extent of surgical margin in the post-NACT setting is a matter of continuing debate. A nonconcentric tumor shrinkage poses a theoretical possibility of isolated tumor cells being left back while attempting conservative resections, leading to an increased rate of margin positivity or local recurrence [26].

## Conclusion

For oral cancer, surgery is still the primary therapeutic option. To establish surgical adequacy, a wide excision with sufficient margins in all three dimensions must be carried out. Adequate margins in the areas of soft tissue, mucosa, and bone must be achieved in all three dimensions. By administering NACT, some tumors that are on the verge of being surgically excised can become surgically excised.
